# Bacterial Volatile Organic Compounds as Potential Caries and Periodontitis Disease Biomarkers

**DOI:** 10.3390/ijms26083591

**Published:** 2025-04-10

**Authors:** Maisa Haiek, Vladislav Dvoyris, Yoav Y. Broza, Hossam Haick, Ervin Weiss, Yael Houri-Haddad

**Affiliations:** 1 Institute of Dental Sciences, Hadassah School of Dental Medicine, The Hebrew University, Jerusalem 9112102, Israel; houri.yael@mail.huji.ac.il; 2 Independent Researcher, HaHaroshet St. 12, Or Yehuda 6037580, Israel; vladi@dvoyris.com; 3 Faculty of Chemical Engineering, Technion and Russell Berrie Nanotechnology Institute—Israel Institute of Technology, Haifa 3200003, Israel; yybroza@technion.ac.il (Y.Y.B.); hhossam@technion.ac.il (H.H.); 4 The Maurice and Gabriela Goldschleger School of Dental Medicine, Tel-Aviv University, Tel-Aviv 6997801, Israel; ervinw@tauex.tau.ac.il; 5 Department of Prosthodontics, Hadassah School of Dental Medicine, The Hebrew University, Jerusalem 9112102, Israel

**Keywords:** *Streptococcus mutans*, *Porphyromonas gingivalis*, *Fusobacterium nucleatum*, *Streptococcus sanguis*, metabolomics, gas chromatography–mass spectrometry, oral diseases

## Abstract

Oral diseases represent a significant global health and economic burden, necessitating the development of effective diagnostic tools. This study investigates the volatile organic compound (VOC) profiles of bacteria associated with dental caries and periodontal disease to explore their potential as diagnostic biomarkers. Four microbial strains—*Streptococcus mutans* (700610), *Streptococcus sanguis* (NCO 2863), *Porphyromonas gingivalis* (ATCC 33277), and *Fusobacterium nucleatum* (PK1594)—were cultured (*N* = 24), alongside intraoral samples (*N* = 60), from individuals with common oral diseases. Headspace VOCs were analyzed using gas chromatography-mass spectrometry (GC-MS), and statistical analyses were conducted by applying non-parametric Wilcoxon and Kruskal–Wallis tests. VOC identification was performed using the NIST14 database. Strain-specific VOC signatures were identified, with *P. gingivalis* and *F. nucleatum* exhibiting distinct profiles from each other and from *Streptococcus* strains. Comparative analysis of disease cohorts revealed statistically significant differences at multiple retention times between caries, gingivitis, and periodontitis. These findings suggest that VOC profiling enables differentiation between bacterial strains and disease phenotypes, supporting their potential application as diagnostic biomarkers for oral diseases. This study establishes a foundational framework for VOC-based diagnostic methodologies in dental pathology.

## 1. Introduction

An estimated 3.5 billion people worldwide suffer from oral diseases [1]. Dental caries and periodontal disease, the most prevalent oral disorders, collectively present 2–2.5 billion [1,2] and 1 billion cases [1], respectively. Research on their underlying causes has evolved over time. The prevailing “Ecological Plaque Hypothesis” by Marsh [3,4] suggests that dental caries result from an imbalance in the microbial community favoring pathogenic species under ecological stress. Kleinberg [5] and Takahashi’s [6] extension of Marsh’s hypothesis emphasized the contribution of acidogenic/aciduric bacteria to the caries process, in which an acidic environment is established by initial tooth colonizers, mainly *Streptococcus sanguinis*, *Streptococcus oralis*, and *Streptococcus mitis* [7]. This triggers a shift in the neutral microenvironment of the plaque towards acid-induced selection and adaptation of the microflora, where mutans streptococci and other aciduric bacteria can flourish under sugar-metabolism-induced acidic conditions [8,9]. These circumstances lead to the dissolution of hydroxyapatite crystals from the tooth surface, contributing to the development and progression of dental caries [10].

In early stages of periodontitis, anaerobic Gram-negative species, including *Fusobacterium nucleatum* (*F. nucleatum*), colonize the tooth surface. *F. nucleatum* plays a critical role in bridging the formation of dental plaques [11,12] through its Fusobacterium adhesionA (FadA) molecule, enabling its attachment to host cells and other bacteria such as *Porphyromonas gingivalis* (*P. gingivalis*) to assist in its colonization [9]. As the disease advances, late pathogenic colonizers, including *Tannerella forsythia*, *Tannerella denticola*, and *P. gingivalis* [13], replace the early colonizers, modulating local immune responses and producing virulence factors that damage the tooth and surrounding bone structures [9,14,15,16]. Previous studies have demonstrated that metabolic activities of host cells, diseased cells, and microbial cells result in the release of endogenous organic volatiles, functioning as metabolic byproducts or signaling molecules [17,18]. Volatilomics has enabled comprehensive profiling of metabolic signatures, aiding in the identification of disease-associated biomarkers and supporting diagnostic advancements in conditions such as neurological disorders, malignancies, and inflammatory diseases [18,19]. VOCs include diverse chemical classes such as fatty acids, hydrocarbons, alcohols, ketones, nitrogen-containing compounds, and volatile sulfur compounds [20]. Due to their low molecular weight (<300 Da), high vapor pressure, and low boiling point, they are volatile and readily excreted into biological samples like breath, urine, and tissues at trace concentrations (ppb or ppt) [21,22,23]. Gas chromatography-mass spectrometry (GC-MS) is the gold standard for VOC analysis, separating compounds based on volatility and interactions with the stationary phase. Retention time, a key parameter, aids in preliminary identification by reflecting the time a compound takes to traverse the column. Variations in chemical properties result in distinct retention times, facilitating differentiation before mass spectral analysis. Ionized and fragmented compounds generate characteristic mass spectra which, when compared with spectral libraries and reference standards, enable precise VOC identification and characterization, along with insights into their biological significance [24].

Although VOCs have been extensively studied as biomarkers for systemic diseases, their role in oral health has garnered limited attention. The presence of oral VOCs may confound systemic disease analyses, necessitating the establishment of standardized protocols for identification to mitigate interference. Furthermore, diverse oral bacteria produce various VOCs; however, the variability in VOC profiles between non-pathogenic and pathogenic oral disease bacteria remains uncertain. As far as the authors are aware, to date, in the realm of oral research, the predominant focus has been on analyzing VOCs emitted from saliva, urine, skin emanations, and exhaled breath. Limited attention has been directed towards exploring the oral biofilm and biomatrices associated with carious dentine. Also, studies have predominantly concentrated on the non-specific, non-targeted analysis of volatile compositions in the oral cavity and sulfur-containing compounds linked to conditions such as halitosis and periodontal diseases [25,26,27,28,29,30,31,32,33,34]. Investigations concerning VOCs in relation to other oral conditions like head and neck cancers are limited, while common oral diseases such as caries and periodontal diseases have received scant consideration, if any [35,36,37,38,39,40]. The lack of clinical correlation between VOCs and under-investigated, prevalent oral health conditions is attributed to the aligned focus on in vitro investigations instead of clinical settings. Translational research is necessary to validate the clinical efficacy of VOCs for diagnosing oral diseases, especially due to the persisting diagnostic limitations that could lead to misdiagnoses, inadequate treatments, and inaccurate assessments of disease prevalence [41,42].

We hypothesized that distinct cariogenic and periopathogenic bacterial strains produce discernible variations in VOC profiles, and such discrepancies will be evident in VOC profiles of intraoral swab samples from individuals with gingivitis, periodontitis, and caries. The primary aim is to investigate bacteria-specific and disease-specific volatile profiles. These were assessed through non-targeted GC-MS analysis of volatile headspace emitted from in vitro cell cultures of cariogenic and periopathogenic bacterial strains. Subsequently, cultured intraoral swab samples obtained from individuals afflicted with gingivitis, periodontitis, and caries were analyzed to establish correlations between VOC profiles and oral diseases. By transitioning stock bacterial strains to patient-derived samples, this study enhances the clinical relevance and facilitates translation to diagnostic clinical settings.

## 2. Results

### 2.1. Stage I

**Figure 1:**
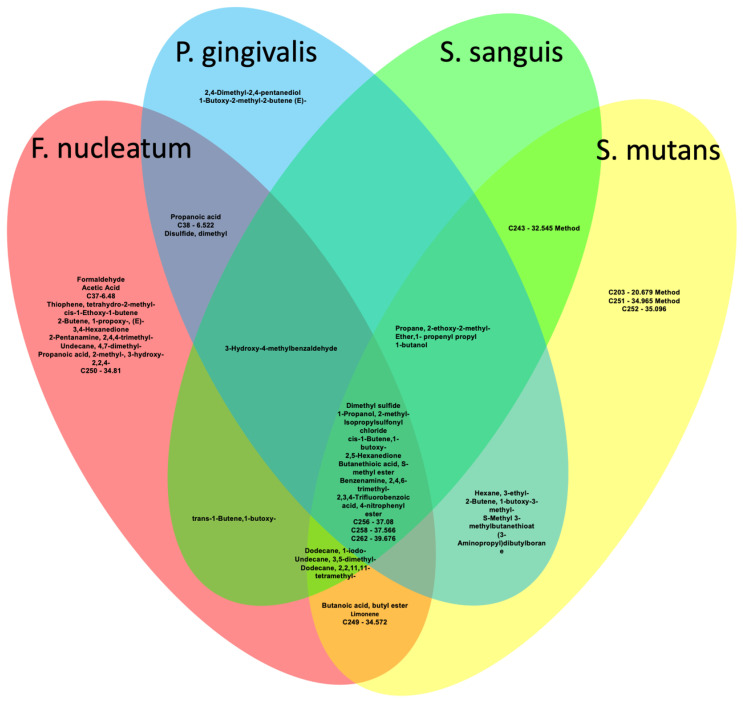
Venn diagram of signature VOCs of *Fusobacterium nucleatum*, *Porphyromonas gingivalis*, *Streptococcus sanguis*, and *Streptococcus mutans*.

A total of 228 volatiles were detected in the headspaces above microbial strains. Among these, 181 compounds were found to be present in both control and sample headspaces, with differences observed in their relative concentrations based on chromatographic peak areas. However, 47 materials (20.6%) were exclusive to sample headspaces. Each bacterial strain characterized by variations in molecule types and chromatograph peak sizes, as measured by the peak areas, demonstrated a unique VOC signature, with *F. nucleatum* displaying 34 molecules, *P. gingivalis* 24 molecules, *S. mutans* 28 molecules, and *S. sanguis* exhibiting 21 molecules, as illustrated in Table 1 and Figure 1.

### 2.2. Stage II

A total of *N* = 60 intraoral samples (caries n *n* = 22, gingivitis *n* = 19, periodontitis *n* = 19) were investigated. Descriptive statistics of the results of Fisher exact tests assessing sex distribution differences among the groups and Kruskal–Wallis tests assessing age differences showed that the average age participants was 49.18 years (SD = 18.20). Among the samples, 56.3% were from females and 43.8% were taken from males. The results indicated a similar distribution of males and females across the groups (*p* = 0.152). However, the intraoral specimens of participants with caries or gingivitis were from a significantly younger age group compared to participants with Periodontitis (*p* < 0.001).

Statistical analyses were conducted to assess the differences in relative peak areas based on retention time across different oral disease cohorts. The GC-MS analysis yielded a total of 3011 chemicals that were detected in at least 1% of the samples. A total of 51 compounds underwent testing. To establish the relevance of the VOCs, those present in fewer than 70% of the samples were considered too infrequent for reliable statistical analysis and were excluded. This resulted in 19 VOCs being considered. Compounds detected in both the control and sample groups were excluded, six VOCs remained that were found to be significant. The headspaces of the studied cultures contained compounds such as carboxylic acid, aldehyde, alkane, carbonic acid, and cyclic compounds. Kruskal–Wallis tests were employed to assess variations in distribution among the cohorts. Subsequent Mann–Whitney pair-wise tests revealed statistically significant differences between groups at multiple retention times, as outlined in Table 2.

The Kruskal–Wallis test was employed as a non-parametric method to determine statistical significance, with KW p denoting the corresponding *p*-values for the test. Subsequently, in cases where significant differences were observed, post hoc Mann–Whitney U tests were performed for pairwise comparisons involving the “caries” and “periodontitis” groups with the “gingivitis” group.

MW p represents the *p*-values obtained from the post hoc test. For all comparisons, a *p*-value of 5% was adopted as the threshold for statistical significance. The number within the [] symbol signifies the count of samples in which each compound is present.

**Figure 2:**
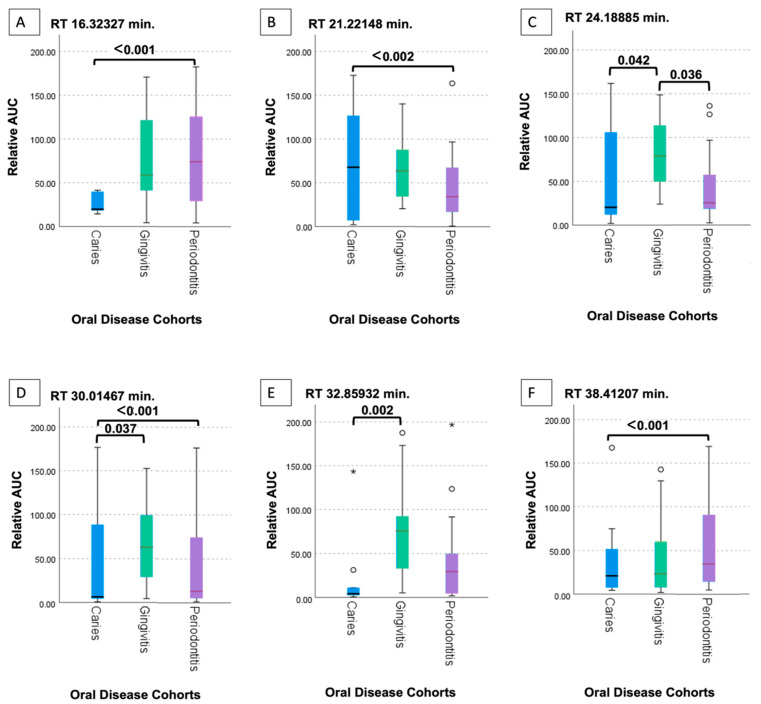
Box-plots illustrating a comparative analysis of volatile organic compounds (VOCs) observed in cultured intraoral clinical samples of common oral diseases, considering only compounds with a cut-off of 70% or above, and excluding room control or empty tubes. (**A**) RT 16 32327. (**B**) RT 21.22148. (**C**) RT 24.18885 (**D**) RT 30.01467 (**E**) RT 32.85932 (**F**) RT 38.41207. Each box-plot corresponds to a specific compound and is associated with its respective retention time (RT), illustrating the relative abundance of the compounds quantified by the area under the curve (AUC). The median of the data is represented by the black line within each box. Outliers are displayed as follows: ◦ = Mild outlier (1.5 to 3 times the interquartile range [IQR] from Q1 or Q3); * = Extreme outlier (greater than 3 times the IQR from Q1 or Q3). The whiskers extending from the upper to lower quartiles provide a visual representation of the variability in the data.

Specifically, noteworthy distinctions were observed between the caries and gingivitis groups at retention times RT = 24.18 (*p* = 0.042), RT = 30.01 (*p* = 0.037), and RT = 32.85 (*p* = 0.002). Additionally, a significant difference was identified between the periodontitis and gingivitis groups at retention time 24.18 (*p* = 0.036), and between caries and periodontitis at RT = 16.32 (*p* ≤ 0.001), RT = 21.22 (*p* ≤ 0.002), RT = 30.01 (*p* ≤ 0.001), and RT = 38.41 (*p* ≤ 0.001) (Figure 2A–F). Utilizing the NIST14 database, a total of six VOCs were tentatively identified (16.32—Propanoic acid 2-2dimethyl (isobutyric acid), 21.22—Benzaldehyde, 24.18—Undecane 2-methyl, 30.01-1-iodododecane, 32.85-Carbonic acid, 38.41—Phenyl-4-azafluorenone, or Ethylene brassylate, or 9,10-Phenanthrenedione).

The statistical significance observed in the Kruskal–Wallis test for the various compounds is demonstrated in Figure 2A–F. Specifically, significant post hoc comparisons using the Mann–Whitney U test were observed between the gingivitis and caries groups in Figure 2C (*p* = 0.042), D (*p* = 0.037), and E (*p* = 0.002). Figure 2A (*p* ≤ 0.001), B (*p* ≤ 0.002), D (*p* ≤ 0.001), F (*p* ≤ 0.001) show the comparison of caries and periodontitis. Additionally, only the compound represented in Figure 2C (*p* = 0.036) showed significance when comparing the periodontitis and gingivitis groups. These findings highlight the potential discriminatory power of VOCs in distinguishing between different oral disease states.

## 3. Discussion

This exploratory study identified significant differences in VOC profiles across bacterial strains and clinical groups. Each strain exhibited unique compounds spanning multiple chemical classes, including aldehydes, carboxylic acids, sulfur compounds, ethers, esters, ketones, and hydrocarbons. Table 1 presents barcode-like VOC patterns, supporting species-specific profiles absent in controls. These findings align with previous studies [20,37,40], including that of Hertel et al. [35], which analyzed volatile profiles of cariogenic bacteria *S. mutans*, *L. salivarius*, and *P. acidifaciens* by GC-MS, detecting 64 unique compounds.

Distinct volatile profiles were noted among strains. *P. gingivalis* produced two unique compounds: 1-Butoxy-2-methyl-2-butene (ether alcohol) [43,44] and 2,4-Dimethyl-2,4-pentanediol [45]. *F. nucleatum* exhibited formaldehyde and C250—34.81, while *S. mutans* produced C203—20.679, C251—34.965, and C252—35.096. Notably, formaldehyde and methyl mercaptan have been linked to head and neck squamous cell carcinoma (HNSCC) and primary lung cancer [46,47,48,49]. While these compounds have known metabolic origins, the role of *Fusobacterium* in formaldehyde production remains unclear [50,51].

Furthermore, *P. gingivalis* was found to produce dimethyl disulfide (DMDS), a compound associated with halitosis and malodor produced by bacteria within the gastrointestinal and respiratory tracts and oral cavity [20,32,51,52,53]. This aligns with findings by Roslund et al. [37] who identified a mass signal at *m*/*z* 95 attributed to phenol or DMDS in the headspace of *P. gingivalis, T. forsythia*, *P. intermedia*, and *P. nigrescens* using PTR-TOF-MS. DMDS production by *P. gingivalis* warrants further investigation into its metabolic pathways and potential clinical relevance [54,55].

Acidic conditions may enhance VOC release. For instance, *F. nucleatum* produces carboxylic acids, such as acetic and propanoic acid. Takahashi et al. [53] showed that pH significantly influences bacterial growth and metabolism. *F. nucleatum* thrives in a pH range (5.5–7.0) and efficiently metabolizes glucose, while *P. gingivalis* requires a pH above 6.0 and shows limited glucose utilization. These pH-dependent metabolic activities may contribute to bacterial proliferation, VOC production, and pathogenic potential, linking microbial behavior to oral health and disease.

To explore the diagnostic potential of VOCs, the authors analyzed the oral microbiome of individuals clinically diagnosed with caries and periodontal phenotypes. The primary aim was to identify multivariate biomarker patterns, serving as proof of concept for diagnostic translation. Figure 2A–F illustrates VOC comparisons among disease phenotypes. The inclusion of a “gingivitis” group held particular significance in the context of the research objectives, as it served to distinguish between a VOC profile indicative of oral health and one associated with oral disease. Six VOCs were tentatively identified: 16.32—Propanoic acid 2-2dimethyl (isobutyric acid), 21.22—Benzaldehyde, 24.18—Undecane 2-methyl, 30.01—1-iodododecane, 32.85—Carbonic acid, and 38.41—Phenyl-4-azafluorenone, or Ethylene brassylate, or 9,10-Phenanthrenedione. Two of these compounds, identified at RT 16.32 and RT 32.85, are likely of biologic origin.

Periodontal disease is associated with short-chain fatty acids (SCFAs) produced by pathogenic bacteria. SCFAs, including acetic acid, propionic acid, butyric acid, and isobutyric acid, have antimicrobial properties that influence microbial composition [56]. One study used GC-MS to determine the kinds and amounts of SCFAs in saliva and bacterial cultures of periodontopathogens (*P. gingivalis* (ATCC 33277) and *F. nucleatum). They compared samples* from 11 severe periodontal disease patients and 10 healthy controls and found that patients with severe periodontal disease patients had elevated levels of salivary butyric, isobutyric, isovaleric, and propionic acid compared to healthy controls [56,57]. Additionally, SCFAs affect oral epithelial cell viability by inducing pyroptosis and apoptosis, disrupting epithelial integrity, and influencing immune responses [44,58].

Carbonic acid and bicarbonate play a role in caries formation by modulating pH levels. An increase in bicarbonate concentration raises salivary pH, potentially affecting the virulence of cariogenic bacteria. The relationship between pH and bicarbonate concentration follows the Henderson–Hasselbalch equation, pH = pK + log[HCO_3_^−^]/[H_2_CO_3_], where pK (6.1) and [H_2_CO_3_] (1.2 mmol/L) are unaffected by flow rate [59].

External factors, such as diet, oral hygiene, and exposure to external chemicals may alter VOC composition, introducing variability that complicates biomarker identification and increases the risk of false positives. The hosts’ genetic predisposition and host-microbiome interactions, such as inflammation and immune responses, contribute to variations in VOC production and can also influence VOC profiles and complicate interpretation. The intraoral biofilm VOCs may obscure individual species’ contributions due to polymicrobial interactions. Synergistic and antagonistic microbial relationships, as well as overlapping metabolic pathways between different microbial species, can produce similar VOCs, reducing specificity in distinguishing between pathogens. Further challenges include the masking effect, where dominant VOCs overshadow others, making detection difficult. Additionally, culturing methods require specific conditions (temperature, pH, growth environment, etc.) that deviate from replicating the natural environment of the oral cavity. Culturing may not capture all microbial species in a sample. Many microorganisms are challenging to culture and thus underrepresented in the analysis. Discrepancies between laboratory conditions and the complex oral ecosystem can affect the metabolic activities and VOC production of bacteria, potentially leading to deviations in the observed VOC profiles. While GC-MS remains the gold standard for VOC analysis, its substantial size and the extended duration required for analysis limit its utilization clinically. Advances in analytical techniques, artificial intelligence, and miniaturized detection systems may pave the way for VOC-based diagnostics at the point of care.

## 4. Materials and Methods

**Figure 3:**
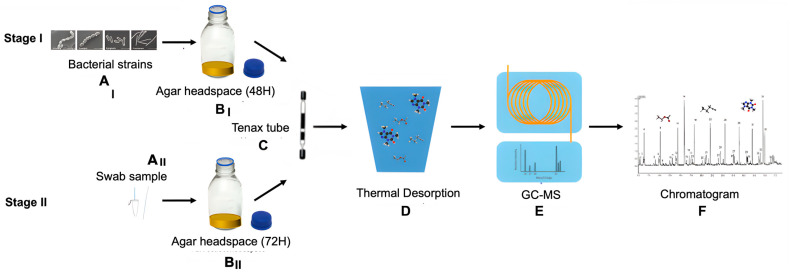
*Stage I*: Four cariogenic and periopathogenic reference microbial strains underwent cultivation (**A_I_**) in hermetically sealed 500 cm^3^ bottles, incubated at 37 °C for 48 h to promote bacterial growth (**B_I_**). The headspaces above the colonies were transferred to adsorbent tubes (**C**). The collected samples were desorbed (**D**) and injected into a GC-MS system (**E**). Statistical analysis was conducted on the peaks and areas under the curve of each sample’s chromatograms (**F**). *Stage II*: Adult participants (*N* = 60) were clinically categorized into three groups—caries, gingivitis, and periodontitis. Microbial specimens were obtained via intraoral swabbing of each participant (**A_II_**). The intraoral samples were cultured in sealed 500 cm^3^ bottles and incubated under anaerobic conditions at 37 °C for 72 h to facilitate microbial growth (**B_II_**). Samples were processed and analyzed under similar conditions as Stage 1.

Experimental Setup: Investigations were performed in a two-stage approach at the Dental Materials Laboratory of the Prosthodontics department, Faculty of Dental Medicine, Hebrew University of Jerusalem, Israel (Figure 3).

Microbiological strain and culture conditions: Four microbial strains (*Streptococcus mutans* (ATCC 700610), Streptococcus sanguis (NCO 2863), *Porphyromonas gingivalis* (ATCC 33277), and *Fusobacterium nucleatum* (PK 1594)) were independently cultured in hermetically sealed 500 cm^3^ bottles, with each strain subjected to 4 independent repetitions. Aerobic and anaerobic strains were cultivated in Brain Heart Infusion (BHI) media (53286-500G Millipore, SigmaAldrich, Jerusalem, Israel) and Wilkins–Chalgren anaerobic agar (W1761, Millipore SigmaAldrich, Israel), respectively. The bottles were incubated at 37 °C for 48 h to allow for bacterial growth.

Intraoral microbiological specimens and culture conditions: Collected intraoral specimens were mixed with 50 cm^3^ of sterile phosphate-buffered saline (PBS) and vortexed, then cultured in sealed 500 cm^3^ bottles containing Brain Heart Infusion Broth (53286-500G Millipore, Sigma-Aldrich) media. In the case of caries samples, a supplement of 3% glucose (Sigma, Rehovot, Israel) was added to the media. All bottles were maintained under anaerobic conditions using AnaeroGen (Thermo Scientific™ Oxoid AnaeroGen 2.5L, Karlsruhe, Germany) and incubated at 37 °C for 72 h.

Inclusion criteria: Participants aged 18 years or older were categorized into three groups based on specific oral diseases: dental caries classified as code 4 or higher under the International Caries Detection and Assessment System (ICDAS) [49]; gingivitis with a healthy periodontium or bleeding on probing < 10% or ≥10%, probing depth < 3 mm, and no radiological bone loss; and periodontitis with bleeding on probing < 10% or ≥10%, probing depth ≥ 5 mm, and radiological bone loss.

Exclusion criteria: Participants with simultaneous presence of multiple distinct oral diseases (e.g., co-occurrence of caries and periodontitis within the same individual), participants diagnosed with cardiovascular disease, inflammatory bowel disease, diabetes mellitus (HbA1c ≥ 7%), ear-nose-throat infections, acute or chronic sinusitis, allergic rhinitis, laryngitis, asthma, chronic obstructive airway disease (COPD), cystic fibrosis (CF), tuberculosis, Parkinson’s disease, Alzheimer’s disease, rheumatic and autoimmune conditions, peptic ulcer disease, gastroesophageal reflux di ease, kidney diseases, previous or active malignancies, former smokers who had quit within the past 5 years or less and active smokers, those with alcohol or drug addiction, pregnant women, and those who had engaged in any oral hygiene activities or consumed food or beverages within the preceding 2 h before sampling were excluded.

Drop-out criteria: Participants who encountered difficulties during the sampling process were withdrawn at the patient level. At the laboratory level, broken Tenax tubes, tube leaks during GC-MS runs, and corrupted files were excluded.

Experimental Procedure:

Stage 1: In Vitro Headspace Collection

For proof of concept, a small sample size (*n* = 4 per bacterial strain) was used to optimize the experimental setup and validate the methodology. Headspaces (*N* = 24) were randomly assigned an order and collected from cultures of *S. mutans*, *S. sanguis*, *P. gingivalis*, and *F. nucleatum*, along with blank (*n* = 4) and aerobic/anaerobic controls (*n* = 6) to minimize bias related to environmental conditions. Samples were transferred to preconditioned adsorbent tubes (Sigma-Aldrich, 28718-U SUPELCOTenax^®^ TA/Carboxen^®^ 1018, Israel), validated for VOC analysis and storage [35].

A butterfly IV cannula (21G) was used to connect the culture bottles to the Tenax tube, while a second hose connected the Tenax tube to a custom-made electric pump. To prevent the ingress of volatile VOCs into the system, a constant flow rate of 10 cm^3^/min for 5 min was utilized based on reports in the literature indicating pump flow rates ranges from 5 to 200 cm^3^/min for tubes with a 1/4-inch diameter, had minimal variation in retention volume despite this wide range. It is worth noting that carboxen sorbents are particularly sensitive to high humidity, which can lead to decreased retention of a wide range of VOCs. Future clinical studies may consider the use of an alternative sorbent material less sensitive to high levels of humidity in the oral cavity [60,61].

The trapped samples were stored at 4 °C until subsequent analysis.

Stage 2: Clinical Sample Collection

A larger cohort (*N* = 60) of adult participants (≥18 years) was included for more extensive analysis following optimization in Stage 1. Participants were recruited from the Dental Student’s Clinic at the Faculty of Dental Medicine, Hebrew University of Jerusalem. Eligible participants were categorized into three groups based on their clinical and radiological assessments: dental caries (*n* = 22), gingivitis (*n* = 19), and periodontitis (*n* = 19). All participants granted written informed consent after approval from the Institutional Review Board (IRB) of the Hadassah Medical Organization (0088-16-HMO). The study adhered to the ethical standards outlined in the Declaration of Helsinki and its subsequent revisions. To meet the International Committee of Medical Journal Editors (ICMJE) requirements, the study was registered at clinicaltrials.gov (ID num. NCT02931032).

Microbial specimens were collected from the oral cavity of each participant. These included samples from infected carious lesions in participants diagnosed with caries, and plaque samples taken from the cervical region of mandibular incisors, for the remaining groups. Samples were incubated for 72 h, and the headspace collection followed the method described in stage I. Empty tubes and room controls served as controls.

Data Collection: Samples were transferred to the Laboratory for Nanomaterial-Based Devices (LNBD) at the Faculty of Chemical Engineering, Technion–Israel Institute of Technology. Tube contents were desorbed (270 °C) and analyzed on a GC-MS system coupled with a thermal desorption device (TD-20-GC2010-GCMS-QP2010 system, Shimadzu Japan). Blank injections were performed between samples to monitor and eliminate any potential carry-over during GC-MS analysis. Additionally, after each GC-MS run, the sorbent tubes were conditioned and purged with nitrogen gas to remove residual analytes. The GC−MS of Agilent (GC-7890B/MS-5977A) was equipped with an SLB-5ms capillary column (with 5% phenyl methyl siloxane; 30 m in length; 0.25 mm internal diameter; 1 μm thickness; purchased from Sigma-Aldrich).

Pre-desorption of the tubes lasted 2 min in a constant N_2_ flow of 20 mL/min, followed by tube desorption under the following conditions: 8 min at 270OC and 40 mL/min N_2_ flow. Cold-trap desorption conditions were 3 min at 270 °C with N_2_ flow of 4 mL/min. The samples were injected automatically from the TD into the GC-system at constant pressure and under 2 mL/min column flow in splitless mode. Two oven temperature profiles were used for two types of analysis. Method 1: Injection with a constant column flow of 2 mL/min. The following oven temperature profile was set: (a) 5 min at 40 °C; (b) 10 °C/min ramp until 300 °C; and (c) 10 min at 300 °C. Method 2: Injection with a constant column flow of 2 mL/min. The following oven temperature profile was set: (a) 10 min at 35 °C; (b) 7 °C/min ramp until 200 °C; (c) 20 °C/min ramp until 300 °C; and (d) 3 min at 300 °C. The peaks and areas under the curve of each sample were subjected to statistical analysis. To enable immediate reuse for further headspace trapping, the sample tubes were conditioned and dry-purged with a constant flow of nitrogen gas (99.999%) through a TC-20 instrument (Markes International Ltd., UK) following the TD-GC analysis [60].

Statistical Analysis: Descriptive statistics of the results of Fisher exact tests assessing sex distribution differences among the groups, and Kruskal–Wallis tests assessing age differences. GC-MS chromatograms for Microbiological strain and Intraoral microbiological specimens were analyzed separately using MassHunter Qualitative Analysis software (version B.07.00, Agilent Technologies, CA, USA) with manual inspection. A semi-targeted approach for VOC identification was applied. The VOCs were identified through mass-to-charge (*m*/*z*) ratio matching using the NIST14 mass spectral library (National Institute of Standards and Technology, MD, USA), allowing for the detection of both pre-characterized and exploratory compounds. This approach aimed to balance known VOC detection with the exploration of new, potentially relevant compounds. In stage I, multiple comparisons were conducted in SAS JMP software (Version 14.0, SAS Institute, Cary, NC, USA) to identify significant differences in VOCs. One-way analysis of variance (ANOVA) and pairwise comparisons using Student’s *t*-test with an LSD threshold Matrix were used for statistical analysis in comparisons between bacterial strains. In stage II, SPSS version 27 was used for data analysis, employing the Kruskal–Wallis test and post hoc Mann–Whitney test for specific pairwise comparisons with gingivitis. VOCs with an observation cut-off of 70% or above were considered, excluding VOCs from empty tubes and room controls. *p* values < 0.05 were deemed statistically significant.

## 5. Conclusions

This study provides empirical evidence supporting distinct VOC molecules and patterns associated with cultured bacterial strains and clinical samples linked to caries and periodontal diseases.

These findings establish a foundational basis for VOC-based diagnostics of common oral diseases while also highlighting putative biomarkers identified based on *m*/*z* ratios and reference library matching that require further validation before clinical application.

Host–microbiome interactions, environmental influences, and the risk of false positives present challenges that must be addressed to enhance diagnostic reliability.

Translational research is essential to validate and implement VOC-based diagnostic approaches in clinical settings, facilitating their practical application in oral health diagnostics.

Future advancements in analytical techniques and machine learning integration may improve the accuracy and feasibility of VOC-based diagnostics for early disease detection.

## Figures and Tables

**Table 1:** Dichotomous analysis of VOC presence or absence for each oral disease-associated bacterial strain. Visualization of VOC profiles in four bacterial strains: *Streptococcus mutans*, *Streptococcus sanguis*, *Porphyromonas gingivalis*, and *Fusobacterium nucleatum*. Highlighted cells indicate strain-specific compounds absent in controls, revealing unique VOC patterns.

Chemical Class	Compound	*F. nucleatum* (34 Molecules)	*P. gingivalis* (24 Molecules)	*S. mutans* (28 Molecules)	*S. sanguis* (21 Molecules)
Aldehyde	Formaldehyde	1			
Carboxylic Acid	Propanoic acid	1	1		
Sulfur Compound	Dimethyl sulfide	1	1	1	1
Ether	Propane, 2-ethoxy-2-methyl-		1	1	1
Carboxylic Acid	Acetic Acid	1			
	C37—6.48	1			
	C38—6.522	1	1		
Alcohol	1-Propanol, 2-methyl-	1	1	1	1
Sulfur Compound	Thiophene, tetrahydro-2-methyl-	1			
Ether	Ether,1-propenyl propyl		1	1	1
Ether	cis-1-Ethoxy-1-butene	1			
Sulfonyl Chloride	Isopropylsulfonyl chloride	1	1	1	1
Alcohol	1-butanol		1	1	1
Diol	2,4-Dimethyl-2,4-pentanediol		1		
Hydrocarbon	2-Butene, 1-propoxy-, (E)-	1			
Ketone	3,4-Hexanedione	1			
Sulfur Compound	Disulfide, dimethyl	1	1		
Amine	2-Pentanamine, 2,4,4-trimethyl-	1			
Hydrocarbon	Hexane, 3-ethyl-		1	1	
Hydrocarbon	trans-1-Butene,1-butoxy-	1			1
Hydrocarbon	cis-1-Butene,1-butoxy-	1	1	1	1
Ketones	2,5-Hexanedione	1	1	1	1
Sulfur Compound	Butanethioic acid, S-methyl ester	1	1	1	1
Ether	trans-1-Butene,1-butoxy-	1			1
Hydrocarbon	2-Butene, 1-butoxy-3-methyl-		1	1	
Thioester	S-Methyl 3-methylbutanethioat		1	1	
Boran Derivative	(3-Aminopropyl)dibutylborane		1	1	
Hydrocarbon	1-Butoxy-2-methyl-2-butene (E)-		1		
Ester	Butanoic acid, butyl ester	1		1	
Terpene	Limonene	1		1	
Aldehyde	3-Hydroxy-4-methylbenzaldehyde	1	1		1
Amine	Benzenamine, 2,4,6-trimethyl-	1	1	1	1
Hydrocarbon	Undecane, 4,7-dimethyl-	1			
Hydrocarbon	Dodecane, 1-iodo-	1		1	1
Hydrocarbon	Undecane, 3,5-dimethyl-	1		1	1
Hydrocarbon	Dodecane, 2,2,11,11-tetramethyl-	1		1	1
Carboxylic Acid	Propanoic acid, 2-methyl-, 3-hydroxy-2,2,4-	1			
	C203—20.679 Method			1	
Carboxylic Acid	2,3,4-Trifluorobenzoic acid, 4-nitrophenyl ester	1	1	1	1
	C243—32.545 Method			1	1
	C249—34.572	1		1	
	C250—34.81	1			
	C251—34.965 Method			1	
	C252—35.096			1	
	C256—37.08	1	1	1	1
	C258—37.566	1	1	1	1
	C262—39.676	1	1	1	1

Notably, the signature of *P. gingivalis* contained two molecules. (1-Butoxy-2-methyl-2-butene (E)-, 2,4-Dimethyl-2,4-pentanediol) unique to this strain, while *F. nucleatum* and *S. mutans* exhibited two (Formaldehyde, C250—34.81) and three unique molecules (C203—20.679 Method, C251—34.965 Method C252—35.096), respectively.

**Table 2:** Results of statistical analysis using non-parametric tests for comparing relative area under the curve and retention time between headspace of cultured intraoral clinical samples of common oral diseases.

RT	Caries Median [N]	Gingivitis Median [N]	Periodontitis Median [N]	KW Statistics Median [N]	KW *p*	MW *p* Caries-Periodontitis	MW *p* Caries-Gingivitis	MW *p* Periodontitis-Gingivitis
16.32	19.64 [6]	58.60 [17]	74.12 [16]	24.82	<0.001	<0.001	0.065	0.958
21.22	67.72 [11]	63.60 [14]	34.25 [17]	29.28	<0.001	0.002	0.371	0.193
24.18	20.27 [12]	78.95 [16]	25.33 [17]	22.24	<0.001	0.922	0.042	0.036
30.01	6.36 [17]	62.89 [16]	13.06 [20]	21.89	<0.001	<0.001	0.037	0.093
32.85	4.11 [9]	75.61 [15]	29.40 [18]	30.99	<0.001	0.102	0.002	0.082
38.41	20.92 [10]	23.09 [16]	34.76 [17]	13.56	0.009	<0.001	0.912	0.271

A summary of the statistical analysis exploring VOC variation across oral-disease profiles. The Kruskal–Wallis test assessed differences in relative peak areas focusing on VOCs present in ≥70% of samples. Key comparisons involved caries, gingivitis, and periodontitis groups, with significant compounds identified based on retention time (RT) values and relative area under the curve (AUC).

## Data Availability

The datasets generated during and/or analyzed during the current study are available from the corresponding author on reasonable request.

## Abbreviations

The following abbreviations are used in this manuscript:

ANOVA	Analysis of Variance
ATCC	American Type Culture Collection
BHI	Brain Heart Infusion
CEET	Collaboration for Environmental Evidence Tool
CF	Cystic Fibrosis
COPD	Chronic Obstructive Pulmonary Disease
DMDS	Dimethyl Disulfide
FadA	Fusobacterium adhesionA
GC-MS	Gas Chromatography-Mass Spectrometry
HbA1c	Hemoglobin A1c
HNSCC	Head and Neck Squamous Cell Carcinoma
ICDAS	International Caries Detection and Assessment System
ICMJE	International Committee of Medical Journal Editors
IRB	Institutional Review Board
LNBD	Laboratory for Nanomaterial-Based Devices
LSD	Least Significant Difference
*m*/*z*	Mass-to-Charge Ratio
NIST	National Institute of Standards and Technology
PBS	Phosphate-Buffered Saline
ppb	Parts per Billion
ppt	Parts per Trillion
PTR-TOF-MS	Proton Transfer Reaction-Time of Flight-Mass Spectrometry
SAS	Statistical Analysis System
SCFA	Short-Chain Fatty Acid
SPSS	Statistical Package for the Social Sciences
TD	Thermal Desorption
VOC	Volatile Organic Compound
